# The association between patterns of early respiratory disease and diastolic dysfunction in preterm infants

**DOI:** 10.1038/s41372-023-01608-5

**Published:** 2023-02-23

**Authors:** Koert de Waal, Edward Crendal, Amy Chin-Yu Poon, Mariyam Shaya Latheef, Elias Sachawars, Thomas MacDougall, Nilkant Phad

**Affiliations:** 1 John Hunter Children’s Hospital, department of neonatology, Newcastle, NSW Australia; 2 University of Newcastle, Newcastle, NSW Australia; 3 John Hunter Hospital, department of cardiology, Newcastle, NSW Australia; 4 John Hunter Hospital, department of radiology, Newcastle, NSW Australia

**Keywords:** Respiratory signs and symptoms, Cardiovascular diseases, Medical imaging

## Abstract

**Background:**

This study aims to determine the association between clinical patterns of early respiratory disease and diastolic dysfunction in preterm infants.

**Methods:**

Preterm infants <29 weeks’ gestation underwent cardiac ultrasounds around day 7 and 14–21. Respiratory dysfunction patterns were classified as stable (ST), respiratory deterioration (RD) or early persistent respiratory dysfunction (EPRD) according to oxygen need. Diastolic dysfunction was diagnosed using a multi-parameter approach including left atrial strain (LAS_R_) to help differentiate between cardiac or pulmonary pathophysiology.

**Results:**

98 infants (mean 27 weeks) were included. The prevalence of ST, RD and EPRD was 53%, 21% and 26% respectively. Diastolic dysfunction was more prevalent in the RD and EPRD groups with patent ductus arteriosus and significant growth restriction as risk factors. Not all infants with a PDA developed diastolic dysfunction. LAS_R_ was lower in the EPDR group.

**Conclusion:**

Respiratory dysfunction patterns are associated with diastolic dysfunction in preterm infants.

## Background

Most very preterm infants require respiratory support early after birth and in the first few postnatal weeks [1]. Three distinct patterns of oxygen requirement and need of respiratory support have been described in this age group [2]. Infants considered stable (ST) require little respiratory support and stabilize quickly, and remain in low fraction of inspired oxygen (FiO_2_) for weeks until they come off respiratory support. Others have higher FiO_2_ at birth and remain oxygen dependent for several weeks, referred to as early and persistent respiratory dysfunction (EPRD). A third group recover from their initial respiratory disease, only to deteriorate in the second week of life, described as a group with respiratory deterioration (RD). The RD or persistent dysfunction has mostly been attributed to pulmonary parenchymal disease with abnormal lung development due to preterm birth. However, some preterm infants have been shown to have early respiratory morbidity due to diastolic dysfunction from immature myocardial development [3, 4].

Clinical trials have targeted very preterm infants with EPRD and RD in the second or third week of life with systemic steroids targeting lung inflammation to prevent further lung disease [5–7]. The results of these trials have only been moderately successful (dexamethasone) or unsuccessful (hydrocortisone), suggesting that some of the infants with EPRD or RD might have an alternative diagnosis. Other pathophysiology that can lead to greater respiratory support requirements in preterm infants in this particular timeframe includes the patent ductus arteriosus (PDA), late onset sepsis and diastolic heart failure (DHF).

Heart failure is defined as an inability of the heart to pump blood to the body at a rate matching its needs, or to do so only at the cost of high ventricular filling pressures [8]. When heart failure presents with normal systolic function, it is classified as DHF. In adults, DHF is characterized by a stiff left ventricle (LV) with decreased compliance and impaired relaxation, which leads to increased end diastolic pressure [9, 10]. Signs and symptoms of DHF are similar to those of heart failure with systolic dysfunction and are created by manifestation of pulmonary edema (RD, exercise intolerance) or systemic oedema. The diagnosis of DHF is supported by identifying diastolic dysfunction with Doppler echocardiography and excluding non-cardiac causes of these signs and symptoms [11].

Up to 1 in 5 very preterm infants develop diastolic dysfunction on cardiac ultrasound in the first weeks after birth [12]. About 10% of those infants continue to develop clinical signs and symptoms that would support the diagnosis of DHF (e.g., RD, systemic oedema), but the clinical signs and symptoms overlap with what we know clinically as RD or EPRD. It is possible that cardiac pathophysiology plays an important role in the development of clinical respiratory dysfunction. The aim of this study is to determine the association between patterns of early respiratory disease and diastolic dysfunction on cardiac ultrasound in very preterm infants.

## Methods

### Patients

This single centre prospective observational study approached all preterm infants <29 weeks’ gestation. Exclusion criteria were significant cardiac or other congenital abnormalities and death before 21 days of life. Infants with a small muscular VSD or small ASD could be included. During the study period local guidelines suggested early surfactant and nasal continuous positive airway pressure via a bubble system as main respiratory support strategy. Management of the PDA was directed by cardiac ultrasound, usually organized in the first week of life. Ethical approval for this study was obtained from the Hunter New England human research ethics committee. After informed consent, all infants received a cardiac ultrasound as close as possible to day 7 after birth, and again between day 14 and 21 after birth. Additional cardiac ultrasounds could be performed at clinicians’ discretion.

### Clinical definitions

Respiratory patterns were determined according to Laughon et al [2]. Infants were categorized as ST when they received FiO_2_ < 0.23 between days 3 and 7 and FiO_2_ < 0.25 at day 14–21, as RD when FiO_2_ < 0.23 between days 3 and 7 and FiO_2_ ≥ 0.25 at day 14–21, and as early persistent respiratory dysfunction (EPRD) when FiO_2_ ≥ 0.23 between days 3 and 7, and FiO_2_ ≥ 0.25 at day 14–21. Respiratory support settings, and blood gas values closest to the cardiac ultrasounds were collected from the patient notes. Where available, chest X-rays were analyzed by two blinded radiologist using pattern recognition on an arbitrary 3 point Likert scale as more likely diffuse or focal neonatal pulmonary disorders, can’t differentiate, or more likely vascular congestion [13].

Weight *Z*-scores were determined using the Fenton growth charts [14]. Significant growth restriction was defined as a birth weight below the 10th percentile or *Z*-score < 1.30. Late onset sepsis was defined as a clinical deterioration with a positive blood culture.

### Echocardiography

Echocardiography examinations were performed using a GE E90 system v202 (GE ultrasound, Illinois, USA) with 12 MHz probe. Image acquisition was triggered to the R wave and followed the recommendations of the American Society of Echocardiography [15]. Analyses were performed offline using Tomtec Arena version 4.6 and 2D Cardiac performance analysis build 1.4.0.44 (Tomtec, Unterschleissheim, Germany). The PDA was assessed from the high parasternal view and the diameter was taken in 2D at end systole. Ejection fraction was determined from the apical 4 chamber view using monoplane method of disk [16].

### Ultrasound parameters of diastolic function

We used a combination of pulse wave Doppler, Tissue Doppler, volumetric changes and speckle tracking imaging to assess diastolic function. Left atrial volume (LAvol) was determined by method of disks from the apical 4 chamber images by tracing the left atrium (LA) at maximum size, omitting the pulmonary vein confluence and the LA appendage [17]. The EA ratio was calculated from the respective early and late diastolic peak velocities from pulsed wave Doppler. The e’ velocity was determined at the septal annulus with tissue Doppler imaging. The Ee’ ratio was calculated from the E wave velocity divided by the e’ velocity.

Peak atrial longitudinal strain was used to help differentiate between diastolic dysfunction of cardiac origin and pulmonary venous hypertension as isolated feature. In isolated pulmonary venous hypertension, one would expect left atrial pressures and function to be normal. Progressive diastolic dysfunction of cardiac origin would increase pressure in all pulmonary vessels (venous, capillary, and arterial). Peak left atrial longitudinal strain was determined at QRS using speckle tracking analysis [18].

Pulmonary hypertension can be the end-result of progressive diastolic dysfunction of cardiac origin, and was assessed by exploring tricuspid regurgitation, moderate to severe septal flattening in systole, or any bidirectional or right-to-left shunt, and recorded as present or not [19].

### Definition of diastolic dysfunction

Diastolic dysfunction was assessed using a multi-parametric approach which included 6 parameters and diagnosed as present when 3 or more parameters were outside the normal range for age. Based on previous work in preterm infants at comparable gestational and postnatal age we set the cut-points at LAvol > 1.50 ml/kg, EA ratio > 0.90, e’ velocity < 2.7, Ee’ ratio > 18, the presence of pulmonary hypertension, and peak left atrial strain <25% [12, 17].

### Statistical analysis

Parameters were expressed as mean and standard deviation or median and interquartile range where appropriate. Simple group analyses were conducted using an independent *t*-test and ANOVA. Categorical variables were expressed as counts (percent) and analyzed using the Chi squared or Fisher’s exact test.

This is an exploratory pilot study with primary outcomes respiratory disease patterns and diastolic dysfunction. An exact sample size could not be calculated due to limited published data on diastolic dysfunction in preterm infants. Assuming that the respiratory patterns are equally divided and 20% develops diastolic dysfunction, a sample of ~100 infants will be required. Known co-variates for respiratory dysfunction and/or diastolic dysfunction include a persistent PDA, mechanical ventilation, late onset sepsis and significant growth restriction [3, 12, 20, 21]. Depending on their prevalence in the studied cohort, actual required sample could vary. Analyses were performed on GraphPad version 6 (Prism, LaJolla, CA, USA) and SPSS version 21 (IBM, Armonk, NY, USA), and *p* values < 0.05 were considered statistically significant.

## Results

From June 2020 to June 2022, 124 preterm infants <29 weeks gestation were admitted in the John Hunter Children’s Hospital neonatal intensive care. Four infants died before 21 days of life, 1 infant was transferred out, 1 infant had significant cardiac abnormalities (large VSD), in 18 cases the investigators were not available and in 2 cases no consent was obtained, leaving 98 infants for analysis. The mean gestational age and birth weight of the cohort was 27.1 (1.4) weeks and 953 (230) grams respectively.

The respiratory patterns and categories are presented in Table 1. The rate of ST, RD and EPRD was 53%, 21% and 26% respectively. Infants with RD or EPRD were generally of lower gestational age and birth weight, and EPRD infants had a lower weight *Z*-score at birth (*p* < 0.05) and were more likely to be male. Diastolic dysfunction was significantly more prevalent in both the RD and EPRD groups. The odds ratio of developing diastolic dysfunction (when compared to the ST group) was 2.2 (95%CI 0.5–9.2) and 3.0 (95%CI 0.8–10.9) on the first scan, and 25 (95%CI 3–225) and 34 (95%CI 4–287) on the second scan for infants with RD and EPRD respectively.

**Table 1:** Patient demographics per clinical respiratory course.

	Stable	Respiratory deterioration	Early persistent respiratory dysfunction	*p* value
*n*	52 (53)	21 (21)	25 (26)	
*Antenatal*				
Pregnancy induced hypertension	14 (27)	3 (14)	9 (36)	0.250
Fetal growth restriction (*Z*-score < −1.00)	7 (13)	4 (19)	8 (32)	0.156
Gestational diabetes	4 (8)	3 (14)	2 (8)	0.658
Prolonged rupture of membranes	9 (17)	8 (38)	5 (20)	0.147
Antenatal steroids	51 (98)	19 (91)	24 (96)	0.332
*Neonatal*				
Birth asphyxia (APGAR < 5 at 5 min)	3 (6)	1 (5)	4 (16)	0.250
Male	26 (50)	10 (48)	19 (76)	0.067
Gestational age	28.0 (0.8)	26.1 (1.4)	26.2 (1.4)	<0.001
Birth weight	1080 (194)	835 (204)	788 (157)	<0.001
Weight *Z*-score	−0.14 (0.71)	−0.12 (0.83)	−0.58 (0.79)	0.017
Small for gestational age (<10th percentile)	2 (4)	1 (5)	3 (12)	0.361
Mechanical ventilation >10 days	0 (0)	4 (19)	9 (36)	<0.001
Patent ductus arteriosus >10 days	5 (10)	8 (38)	13 (52)	<0.001
Late onset sepsis	2 (4)	4 (19)	3 (12)	0.107
Diastolic dysfunction on 1st scan	5 (10)	4 (19)	6 (24)	0.225
Diastolic dysfunction on 2nd scan	1 (2)	7 (33)	10 (40)	<0.001

Data presented as *n*(%) or mean(SD).

**Fig. 1:**
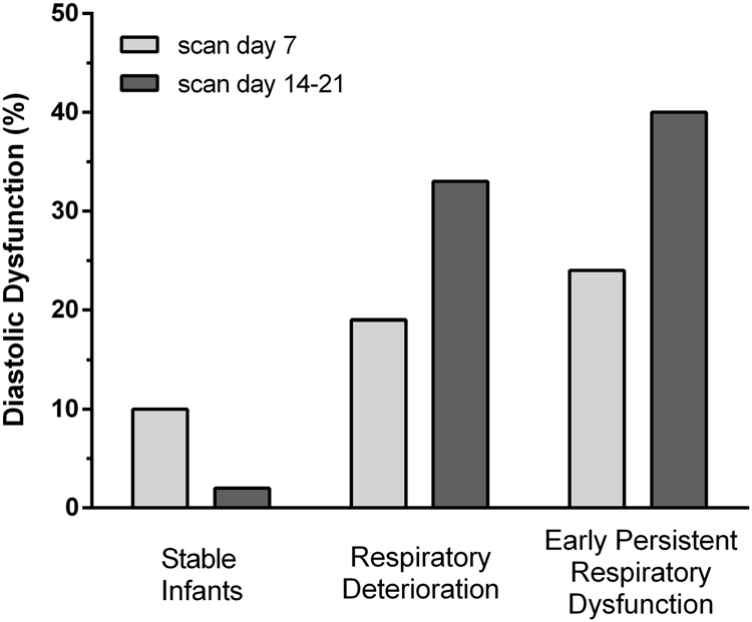
Relationship between patterns of respiratory disease and incidence of diastolic dysfunction on cardiac ultrasound.

**Fig. 2:**
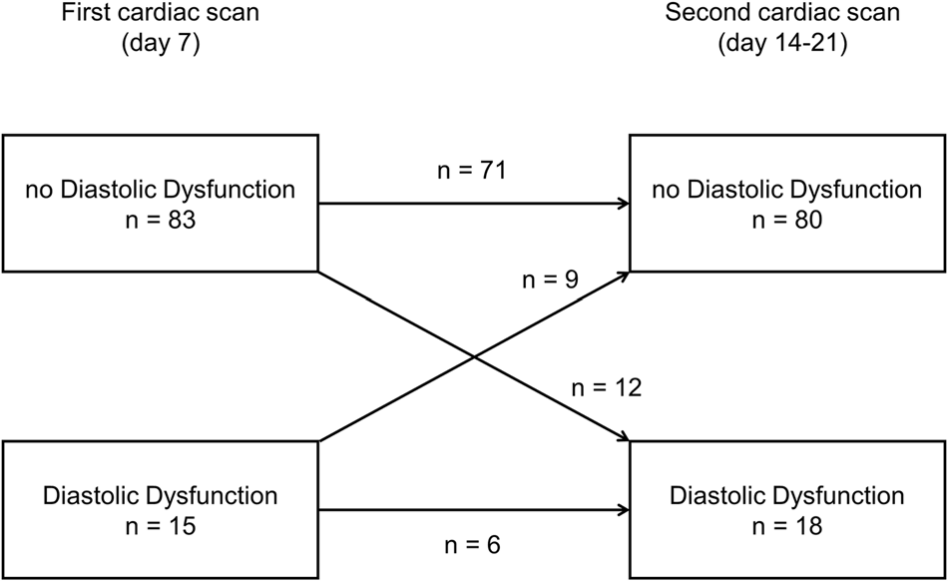
Prevalence of diastolic dysfunction and progression over time.

The first cardiac ultrasound scan was performed at a median of 7 (5–8) days after birth, and the second scan at 18 (15–20) days. Figure 1 shows the prevalence of diastolic dysfunction in relation to respiratory patterns, and Fig. 2 shows its progression over time. Fifteen infants, all with a PDA, had diastolic dysfunction on the first scan. Nine of these improved over time, 5 with concurrent PDA closure. In 12 infants diastolic dysfunction newly appeared at the second cardiac scan, 5 of those had a persistent PDA.

Table 2 shows hemodynamic and cardiac ultrasound parameters for each respiratory group at the second scan. When compared to the ST group, infants with RD and EPRD had lower blood pressure and lower early diastolic e’ velocities at the first scan, but not at the second scan. LAvol and Ee’ ratio was higher at both cardiac scans, with lower atrial strain in the EPRD group only. The prevalence of pulmonary hypertension at the second scan was significantly higher in the RD group (19%) and EPRD group (24%) when compared to the ST group (2%).

**Table 2:** Hemodynamic and cardiac ultrasound parameters.

		First cardiac scan (day 7)			Second cardiac scan (day 14–21)		
		ST	RD	EPRD	ST	RD	EPRD
Heart rate	(beats/min)	163 (11)	164 (11)	169 (12)	162 (8)	163 (7)	165 (10)
Systolic blood pressure	(mmHg)	61 (7)	54 (5)^#^	53 (8)^#^	66 (8)	62 (9)	63 (11)
Diastolic blood pressure	(mmHg)	36 (6)	29 (6)^*^	31 (6)^*^	38 (8)	35 (7)	38 (7)
Ejection fraction	(%)	60 (8)	64 (8)	65 (7)^*^	53 (5)	58 (10)	57 (8)
Left atrial volume	(ml/kg)	0.92 (0.36)	1.41 (0.52)^#^	1.43 (0.70)^#^	1.09 (0.20)	1.53 (0.67)^#^	1.74 (1.22)^#^
EA ratio		0.75 (0.13)	0.81 (0.14)	0.76 (0.17)	0.82 (0.09)	0.85 (0.14)	0.81 (0.15)
e’ velocity	(cm/s)	3.6 (0.6)	3.2 (0.5)^*^	3.1 (0.7)^#^	3.9 (0.4)	3.6 (0.7)	3.9 (1.4)
Ee’ ratio		12.7 (4.2)	17.2 (5.7)^#^	17.3 (8.4)^#^	14.8 (3.1)	18.2 (4.3)^#^	17.9 (6.2)^#^
Peak atrial strain	(%)	32 (7)	30 (7)	29 (8)	32 (4)	29 (6)	27 (8)^*^

Data presented as mean(SD). *t*-test *p* value at ^#^ <0.01 or ^*^<0.05 when compared to the ST group.

*ST* stable group, *RD* respiratory deterioration group, *EPRD* early progressive respiratory dysfunction group.

There were no significant differences at the first or second cardiac scan in sodium, lactate, pH, pCO_2_ and base excess between the groups. Hemoglobin was significantly lower in the RD and EPRD groups at first scan (126(19) and 132(30) versus 147(22) gr/L, *p* < 0.01) and at the second scan (98(17) and 101(16) versus 115(18) gr/L, *p* < 0.01).

Eight infants in the ST group (including the one infant with diastolic dysfunction) received a chest X-ray which were all scored as more likely to represent a pulmonary disorder. A total of 73 chest X-rays were taken in the RD and EPRD groups, with only 2 classified as more likely vascular congestion.

The association between patterns of respiratory disease, diastolic dysfunction and the prevalence of other pathology is presented in supplementary table 1. The prevalence of PDA > 10 days, mechanical ventilation >10 days, significant growth restriction and late onset sepsis was 26%, 13%, 6% and 9% respectively. Diastolic dysfunction in the ST and RD groups was predominantly related to a PDA, and in the EPRD group it was related to growth restriction with or without a PDA. The prevalence of diastolic dysfunction in infants with a persisted PDA > 10 days, mechanical ventilation >10 days, significant growth restriction and late onset sepsis was 46%, 53%, 66% and 33% respectively.

**Fig. 3:**
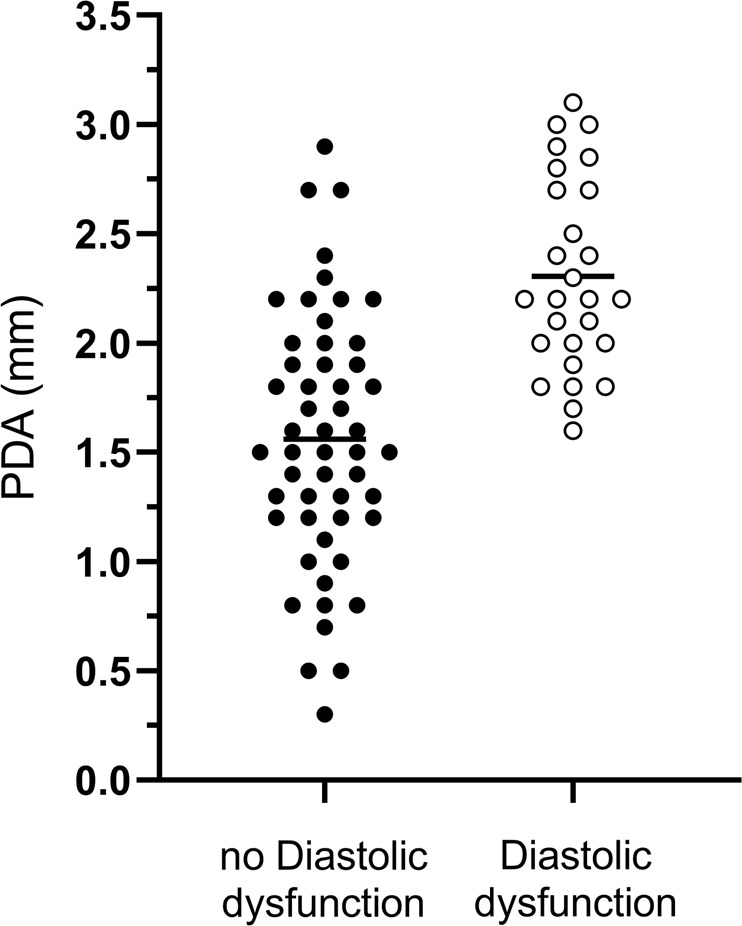
Patent ductus arteriosus (PDA) diameter in relation to diastolic dysfunction.

Figure 3 shows the relationship between the PDA diameter and diastolic dysfunction. Infants with diastolic dysfunction had a significantly larger PDA compared to infants without (2.32 versus 1.56 mm, *p* < 0.001). Not all infants with a large PDA diameter developed diastolic dysfunction.

## Discussion

This exploration study found a strong association between clinical patterns of respiratory dysfunction and the prevalence of diastolic dysfunction on cardiac ultrasound. We hypothesize that cardiac pathophysiology plays an important role in the development of clinical signs and symptoms in a subgroup of preterm infants. Our data showed a strong association between clinical patterns of respiratory dysfunction and the prevalence of diastolic dysfunction on cardiac ultrasound.

Diastolic function of the heart describes the filling of the ventricles. When the mitral valve opens, blood enters the LV through suction caused by elastic recoil of myocardial fibers re-lengthening toward their resting length (restoring forces) and thereby lowering LV cavity pressure. With further filling and increase in LV cavity size, and the myocardial fibers expanding beyond their resting length, stiffness of the myocardial tissues will slow the rate of filling and increase LV cavity pressure. The final phase of diastolic function is created by atrial contraction, increasing the LA pressure above the LV pressure and establishing the final LV end diastolic volume and pressure before the mitral valve closes and contraction begins. Diastolic dysfunction is characterized by an increased pressure at the end of diastole or abnormal patterns or LV myocardial relaxation.

The preterm heart undergoes significant changes with the transition from fetus to newborn, and is exposed to higher preload and afterload than the fetus [22]. The intrinsic differences in cardiac structure and function of the preterm heart when compared to the term heart would make it prone to earlier failure of available compensatory mechanisms. LV afterload might be modulated by altering systemic vascular resistance and lowering blood pressure. PDA closure can assist in reducing preload, but this often does not occur after very preterm birth [23]. Atrial volume and pressure may increase progressively and atrial dysfunction might appear with prolonged periods of increased preload and afterload, often without LV systolic dysfunction [17, 24].

Our current study supports the above-described mechanistic pathways in the development of diastolic dysfunction and associated clinical respiratory signs and symptoms in preterm infants. Blood pressure was lower in the infants with respiratory signs and/or diastolic dysfunction. Atrial volume and the Ee’ ratio was increased in infants with respiratory signs, both parameters associated with raised atrial pressure, and atrial dysfunction was found in the EPRD group where persistent high atrium volume was common. Increased LA pressure can lead to pulmonary venous congestion and a subsequent increase in right ventricle afterload. To compensate the right ventricular driving pressure has to increase, which can explain the higher rate of pulmonary hypertension found in the RD and EPRD groups [4, 25]. Pulmonary hypertension due to pulmonary vascular disease as seen in abnormal preterm lung development would not necessarily increase LA pressure, and thus ultrasound estimates of LA pressure could be proposed to differentiate between a pulmonary or cardiac origin of respiratory signs and symptoms. Chest x-ray was unable to differentiate between respiratory and cardiac diastolic pathophysiology.

The prevalence of diastolic dysfunction was high in infants with respiratory signs and a persistent PDA for more than 10 days, mechanical ventilation for more than 10 days, significant growth restriction and late onset sepsis. The PDA is a hemodynamic condition where high volumes of blood pass through an open shunt into the left side of the heart, leading to progressive morphological and functional changes in the LA and the LV [26]. Besides PDA closure, limited compensatory mechanisms are available to the preterm heart to control the rising LA pressure with a PDA. The foramen ovale is used to offload some of the raised volume and pressure in the LA, but often fails to do so completely [27]. This explains why the presence of a PDA has strong associations with ultrasound parameters of diastolic dysfunction [12, 28–30]. Somewhat unexpectedly, we found that not all infants with a PDA developed diastolic dysfunction despite estimates of high shunt volume. The PDA diameter was not a good predictor of clinical respiratory dysfunction, and we would recommend adding the presence of diastolic dysfunction to select patients for treatment.

Mechanical ventilation and respiratory signs and symptoms are inherently associated with each other, but the relationship between mechanical ventilation and diastolic dysfunction is less well understood. Cyclic changes in intrathoracic pressure can theoretically enhance early diastolic recoil forces [31]. Some studies in preterm infants did not find a relationship early after birth, but Bussmann et al. showed that LV diastolic dysfunction was independently associated with a higher risk of needing mechanical ventilation [3, 32, 33]. Studies in adults have shown that ultrasound parameters of early diastolic function (E wave, e’ velocity, Ee’ ratio) can predict difficulties with weaning off mechanical ventilation [34]. This finding would suggest that diastolic dysfunction drives the need for mechanical ventilation as opposed to mechanical ventilation causing diastolic dysfunction.

Late onset sepsis was more common in infants who developed respiratory dysfunction, but we were unable to establish a significant association with concomitant diastolic dysfunction. Late onset sepsis in preterm infants is usually linked with high blood flows, and one would expect similar pathophysiology in diastolic function as with a PDA [20, 35].

We found that a lower birth weight *Z*-score contributed to persistent respiratory problems and diastolic dysfunction. Intrauterine growth restriction is known for abnormalities in cardiac structure and function that can persist into adulthood [36]. SGA hearts after birth show signs of cardiac remodeling with hypertrophy and dilatation. Altered function can be seen in ultrasound parameters that are associated with myocardial relaxation (low e’ velocity, prolonged relaxation time) or stiffness (shorter E wave deceleration) [37–40]. Diastolic dysfunction in SGA infants appears to occur during relative normal blood flow volumes. This might support the concept that diastolic dysfunction has different phenotypes in preterm infants, similar to what is found in adults [11]. In some infants diastolic dysfunction is created by high volume throughput (PDA, sepsis), and in others it might be primarily caused by abnormal cardiac relaxation or increased myocardial stiffness (SGA).

There are several limitations to the findings of our pilot exploration study. Based on the actual prevalence of some co-variates in this cohort, we acknowledge that our sample is not large enough to provide strong associations between all subgroups. Disparity between patient populations, postnatal age, used ultrasound hardware and analysis software can lead to variation in reference values used to establish diastolic dysfunction. We recommend establishing local reference values to validate a multi-parameter approach to diastolic dysfunction. There remains a significant lack of normative data of ultrasound parameters of diastolic function in preterm infants at various gestation and postnatal age. Our data should not be used to infer judgment on the relationship of diastolic function, treatments for it, and closure effects in the setting of a PDA. This would require a prospective study with selection of patients with truly pathologic shunts.

## Conclusion

Our data showed a strong association between clinical patterns of respiratory dysfunction and the prevalence of diastolic dysfunction on cardiac ultrasound. A multi-parameter approach was able to assess diastolic function and estimate LA pressure. Estimates of high LA pressure could be used to differentiate between cardiac or pulmonary pathophysiology of clinical respiratory signs. The findings in this study can be used to establish the clinical diagnosis of DHF and help select very preterm infants for future studies that target possible treatments to the principal pathophysiology.

### Supplementary information

Supplementary Table 1

## Data Availability

The datasets generated during and/or analyzed during the current study are not publicly available due to patient confidentiality of individual cases, but are available from the corresponding author on reasonable request.
