# Supplementary material for: The association between patterns of early respiratory disease and diastolic dysfunction in preterm infants
Source: J Perinatol. 2023 Feb 23;43(10):1268–73. doi: 10.1038/s41372-023-01608-5 (PMC10541326; doi:10.1038/s41372-023-01608-5)
Supplement: Supplementary file 1 — Supplementary Table 1 [file 41372_2023_1608_MOESM1_ESM.docx]

Supplementary table 1. Association between patterns of respiratory disease, diastolic dysfunction and other pathology at day 14-21. Data presented as number of infants. Chi square p-value *< 0.05 when compared to no diastolic dysfunction.

|  | Stable | | Respiratory deterioration | | Early persistent respiratory dysfunction | |
| --- | --- | --- | --- | --- | --- | --- |
|  | no diastolic dysfunction | diastolic dysfunction | no diastolic dysfunction | diastolic dysfunction | no diastolic dysfunction | diastolic dysfunction |
| n | 51 | 1 | 14 | 7 | 15 | 10 |
| Patent ductus arteriosus > 10 days | 4 | 1* | 3 | 5* | 7 | 6 |
| Mechanical ventilation > 10 days | 0 | 0 | 2 | 2 | 4 | 5 |
| Significant growth restriction | 2 | 0 | 0 | 1 | 0 | 3* |
| Late onset sepsis | 2 | 0 | 3 | 1 | 1 | 2 |
| None of the above pathology | 44 | 0 | 8 | 1 | 5 | 0 |
